# Supplementary material for: Identification of photoperiod-induced specific miRNAs in the adrenal glands of Sunite sheep (Ovis aries)
Source: Front Vet Sci. 2022 Jul 22;9:888207. doi: 10.3389/fvets.2022.888207 (PMC9354845; doi:10.3389/fvets.2022.888207)
Supplement: Supplementary file 1 [file Data_Sheet_1.ZIP › Supplementary file/miRDeep2 file.zip!/miRDeep2 file/Know_Structure/LP42A2/oar-mir-493.pdf]

miRBase precursor : oar-mir-493
Total read count : 8206
oar-miR-493-5p read count : 4684
oar-miR-493-3p read count : 3522
remaining reads : 0

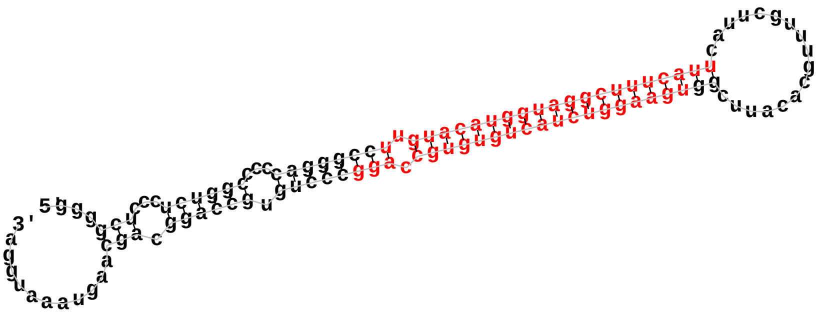

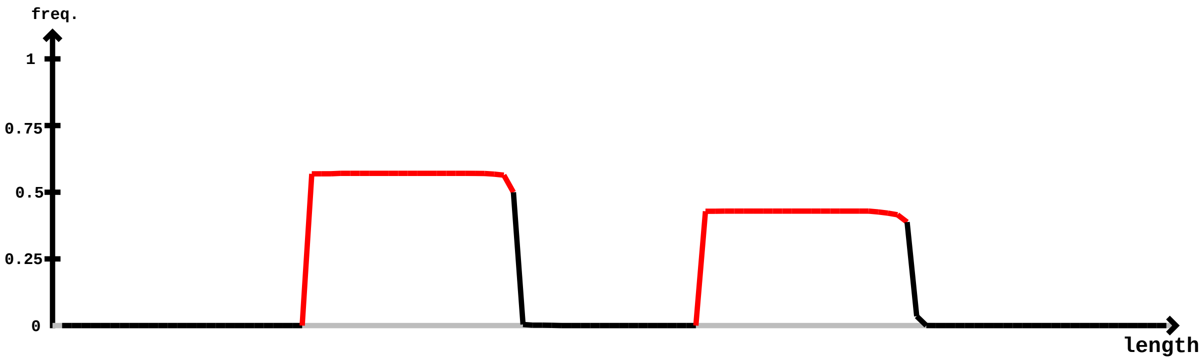

oar-miR-493-5p

oar-miR-493-3p

```
5'- ggggcucccucuggcccccagggccuuguacaugguaggcuuucauucauucguuugcacauucggugaaggucuacugugugccaggcccugugccaggcagcaaguaaaugga   -3'    exp
    ...(((...((((((...((((((((.((((((((((((((((((((.................)))))))))))))))))))).)))))))).)))))).)))...........    reads      mm         sample
    .........................uuguacaugguaggcuu.........................................................................    2          0          seq
    .........................uuguacaugguaggcuuu........................................................................    3          0          seq
    .........................uuguacaugguaggcuuuc.......................................................................    20         0          seq
    .........................uuguacaugguaggcuuuca......................................................................    28         0          seq
    .........................uugCacaugguaggcuuuca......................................................................    3          1          seq
    .........................uuguacaugguaggcuuucau.....................................................................    527        0          seq
    .........................uuguacaugguaggcuuucauu....................................................................    4064       0          seq
    .........................uuguacaugguaggcuuucauuc...................................................................    21         0          seq
    ..........................uguacaugguaggcuuucauu....................................................................    2          0          seq
    ...........................guacaugguaggcuuucauu....................................................................    1          0          seq
    ............................uacaugguaggcuuucauuc...................................................................    1          0          seq
    ............................uacaugguaggcuuucauucau.................................................................    5          0          seq
    ............................uacaugguaggcuuucauucauu................................................................    7          0          seq
    ..................................................................ugaaggCcuacugugugc...............................    1          1          seq
    ..................................................................ugaaggucuacugugugU...............................    9          1          seq
    ..................................................................ugaaggucuacugugugG...............................    3          1          seq
    ..................................................................ugaaggucuacugugugc...............................    13         0          seq
    ..................................................................ugaaggucuacugugugcc..............................    36         0          seq
    ..................................................................ugaagguUuacugugugcca.............................    1          1          seq
    ..................................................................ugaaggucuacugugugcca.............................    50         0          seq
    ..................................................................ugaaggucuacugugugccag............................    219        0          seq
    ..................................................................ugaaggucuacuAugugccag............................    1          1          seq
    ..................................................................ugaaUgucuacugugugccag............................    1          1          seq
    ..................................................................ugaaggucuGcugugugccag............................    4          1          seq
    ..................................................................ugaagguUuacugugugccagg...........................    1          1          seq
    ..................................................................ugaagAucuacugugugccagg...........................    3          1          seq
    ..................................................................ugaaggucuacuAugugccagg...........................    1          1          seq
    ..................................................................ugaaggucuGcugugugccagg...........................    10         1          seq
    ..................................................................ugaaggucuacugugugccagg...........................    2868       0          seq
    ..................................................................ugaaggCcuacugugugccagg...........................    12         1          seq
    ..................................................................ugaaUgucuacugugugccagg...........................    1          1          seq
    ..................................................................ugaaggucuGcugugugccaggc..........................    3          1          seq
    ..................................................................ugaaggucuacugugugccaggc..........................    279        0          seq
    ..................................................................ugaaggucuacugugugccaggcc.........................    1          0          seq
```

```
                                 oar-miR-493-5p
                                                                                     oar-miR-493-3p
ggggcucccucuggcccccagggccuuguacaugguaggcuuucauucauucguuugcacauucggugaaggucuacugugugccaggcccugugccaggcagcaaguaaaugga

...................................................................gaaggucuacuguguUccagg...........................     1          1          seq
....................................................................aaggucuacugugugccaggc..........................     3          0          seq
....................................................................aaggucuacugugugccaggcc.........................     1          0          seq
```
